# 3D DAS VSP for Coal Seam Exploration: A Case Study from Queensland, Australia

**DOI:** 10.3390/s24082561

**Published:** 2024-04-17

**Authors:** Konstantin Tertyshnikov, Alexey Yurikov, Andrej Bona, Milovan Urosevic, Roman Pevzner

**Affiliations:** Centre for Exploration Geophysics, Curtin University, Perth 6151, Australia; konstantin.tertyshnikov@curtin.edu.au (K.T.); alexey.yurikov@curtin.edu.au (A.Y.); a.bona@curtin.edu.au (A.B.); m.urosevic@curtin.edu.au (M.U.)

**Keywords:** DAS, 3D VSP, reflectivity method, borehole

## Abstract

Seismic methods are extensively used in coal mining for expanding resource discoveries and definition as well as for mine monitoring. However, the use of borehole seismic methods is relatively uncommon due to the high cost of borehole seismic acquisition using conventional downhole tools. The introduction of distributed acoustic sensing (DAS), which uses optical fibres to record seismic data, has dramatically increased the cost-effectiveness of borehole seismic methods. Fibre-optic cables are inexpensive and, once deployed in a borehole, can be abandoned or used later for further monitoring of the subsurface. The case study presented here concerns the use of DAS to record a 3D VSP (vertical seismic profiling) for coal seam exploration in Queensland, Australia. This study trialled effective strategies for deploying cables into boreholes and demonstrated how this technology could be incorporated into the standard coal exploration process. The final processing results produced a high-resolution 3D seismic cube where the coal seams below the basalt cover are clearly identifiable around the boreholes. Permanent installation of the fibre-optic cables into a set of boreholes provides immediate benefits of 3D seismic imaging and can create additional value in utilising these sensors for further discrete or continuous subsurface measurements, including stability monitoring of underground workings and detection of methane accumulations.

## 1. Introduction

Seismic methods, especially surface 2D and 3D methods, are extensively used in the coal industry for resource exploration [1,2,3,4] and, recently, using high-density surveys for resource delineation [5]. Having one of the highest safety standards amongst the mineral industry, coal mining also employs seismic techniques for production monitoring. Seismic methods are sometimes used in time-lapse mode to observe the mining process and detect methane accumulations and gas leakages. In some countries, this is becoming a common practice or even a legal requirement [6,7]. Borehole seismic methods using conventional downhole seismic receivers, on the other hand, are less popular in mining due to the relative cost of borehole seismic acquisition. While several attempts have been made to optimise borehole seismic acquisition and its cost-effectiveness using different sensor designs, borehole seismic methods are still not considered standard tools in the industry. 

Over the past ten years, significant advancements have been achieved in distributed acoustic sensing (DAS) technology. This technology is progressively becoming more and more common in various geophysical applications. This method utilises an optical fibre as a sensor. A recording unit known as an interrogator sends a laser pulse down the fibre and receives the light backscattered by inhomogeneities in the fibre. The interrogator records the phase change of the backpropagated light, resulting from subtle elongations or contractions in the fibre when seismic energy interacts with the cable. Naturally, such measurements are more susceptible to waves that cause strain primarily parallel to the fibre-optic cable, such as P-waves propagating along the cable [8]. This preferential directivity is one of the reasons why DAS is a particularly useful method for downhole seismic applications, since in this receiver geometry, the recorded P-waves are mostly polarised along the direction of the cable. Numerous examples have been published on successful applications of fibre-optic sensing for reservoir monitoring in hydrocarbon exploration and CO_2_ geosequestration (e.g., [9,10,11]). The DAS approach has several advantages that make downhole seismic measurements affordable and effective. In particular, unlike geophones, DAS provides dense spatial sampling along the entire length of a borehole, allowing data recording along the entire borehole at once. This greatly reduces the duration, and hence the cost, of acquisition. Distributed fibre-optic sensors can provide data superior to those acquired with geophones [12]. Additionally, standard telecommunication fibre-optic cables are inexpensive enough to be considered disposable and can be permanently installed in a borehole at little extra cost. These advantages make DAS attractive for mineral exploration, where exploration boreholes are usually shallow and can be easily instrumented with such cables. Despite the apparent benefits of DAS for downhole seismic measurements, there are limited examples of its applications for mineral and coal exploration [13,14,15].

It should be noted that the implementation of DAS for coal exploration is not without its challenges. The depth calibration of borehole DAS data is challenging as the acquisition is normally carried out without real-time correlation of the cable location to depth with gamma-ray measurements. Instead, the following approaches can be used for depth calibration: (1) using locations of geophones (in case of simultaneous acquisitions) and optical attenuation points [16]; (2) using locations of clamps in case a cable is clamped to a tubing [17]; (3) using DAS amplitudes and borehole logs [18,19]. Another challenge is the strong dependence of the optical fibre sensitivity on the incidence angle, as discussed above. This directional sensitivity issue can be mitigated using helically wound cables (HWC) [20]. Furthermore, the signal-to-noise ratio (SNR) of standard telecom DAS cables can sometimes be lower compared to geophones and depends on the cable deployment [21]. However, SNR can be greatly enhanced by using engineered fibres with enhanced backscattering properties [12]. 

Another challenge for all borehole seismic methods in coal applications is complex structures, strong velocity contrasts and high attenuation of seismic signals. We believe that, despite these issues, the potential of borehole DAS in the coal industry for exploration and mine operations should be further explored.

Here we present a case study of 3D DAS vertical seismic profiling (VSP) acquisition at an Anglo American coal mining site in Queensland, Australia. The survey was conducted in 2019 and, at the time, was the first of its kind experiment on the acquisition of 3D VSP with fibre-optic sensors in coal exploration. The experiment probed two deployment strategies for the permanent installation of fibre-optic sensors with low cost and minimal disruption of the general drilling process. The main objective was to image a coal seam at a depth of approximately 400 m using fibre-optic cables cemented in three exploration boreholes. A particular challenge for this study was imaging in the presence of near-surface basalts that greatly degrade the quality of the recorded seismic signal. To address this challenge, we complemented the field experiment with a synthetic study aimed at improving and calibrating the processing of the field data. The high-quality 3D volume obtained as a result of the data processing provides a clear image of the target seam around the boreholes and delivers insights into the optimal employment of DAS for coal exploration.

We begin by describing our field experiment; next, we describe a synthetic study based on the field acquisition parameters that we use to inform the further described data processing and interpretation of the results. The experiment demonstrates the effective utilisation of DAS technology for the acquisition of valuable downhole seismic data. 

## 2. Field Experiment

The DAS 3D VSP experiment was conducted in 2019 at a coal mine in Queensland, Australia. The study area is characterised by the presence of basalts at a relatively shallow depth (70–100 m). It is generally difficult to obtain a reliable image under the basalt cover from conventional 3D surface seismic, as these rocks have very high seismic velocities (relative to sedimentary rocks at similar depths) and significantly scatter seismic energy [22]. This issue can be partially addressed by deploying receivers in boreholes, as the reflected energy travels through the highly reflective basalts only once. As such, this feasibility trial aims to demonstrate how relatively inexpensive DAS downhole seismic measurements help image the coal seams under the basalts. 

**Figure 1:**
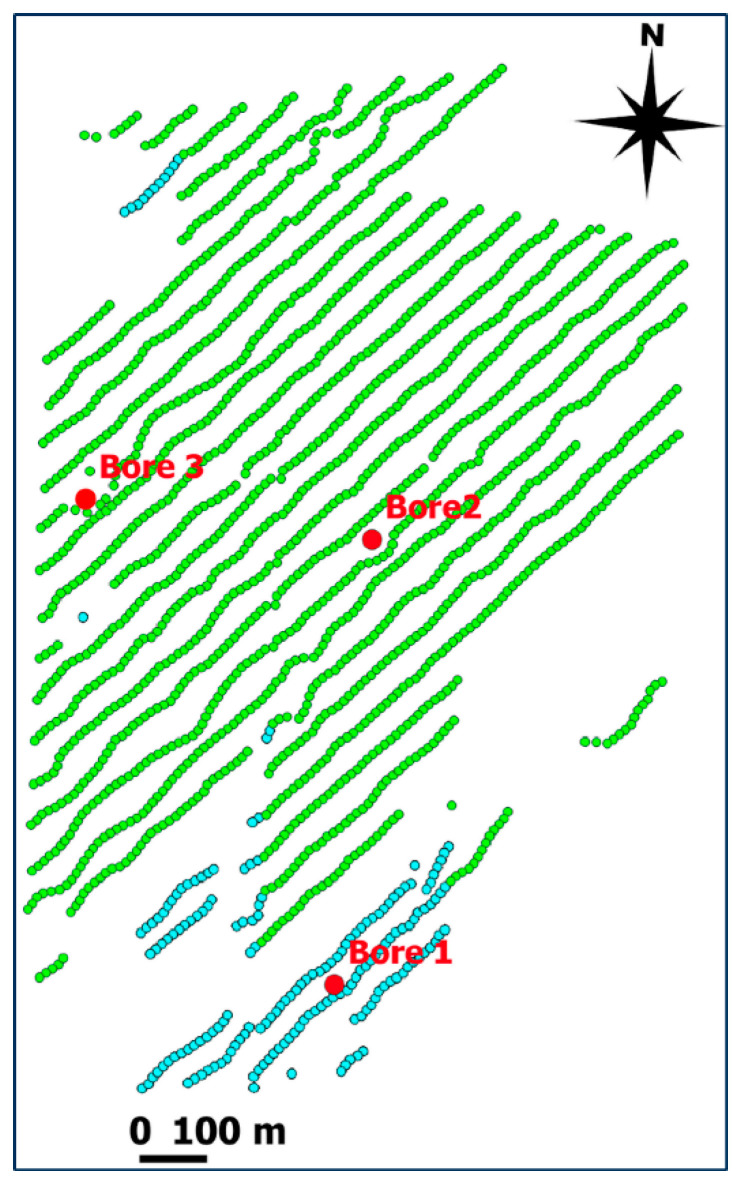
Acquisition map. Red dots show the locations of the boreholes. Green shot points were recorded with fibre optics in Boreholes 2 (east) and 3 (west) (connected together); blue shot points were recorded with fibre optics in Borehole 1 (south).

To reduce the duration and cost of the test, the DAS acquisition was carried out concurrently with a planned surface 3D seismic survey. Three vertical boreholes, originally drilled to sample the target coal seam formation, had fibre-optic cables cemented within them. Each cable had a turnaround at the bottom, which enabled them to be connected together so that data could be recorded using a single interrogator. Figure 1 shows the shot point locations that were acquired with the deployed DAS system. Unfortunately, during the installation, the turnaround in Borehole 1 was damaged, and this borehole needed to be interrogated separately from the other two holes. As such, only a small portion of shot points was acquired with the fibre in Borehole 1 (blue dots in Figure 1), and these data were excluded from further analysis.

**Figure 2:**
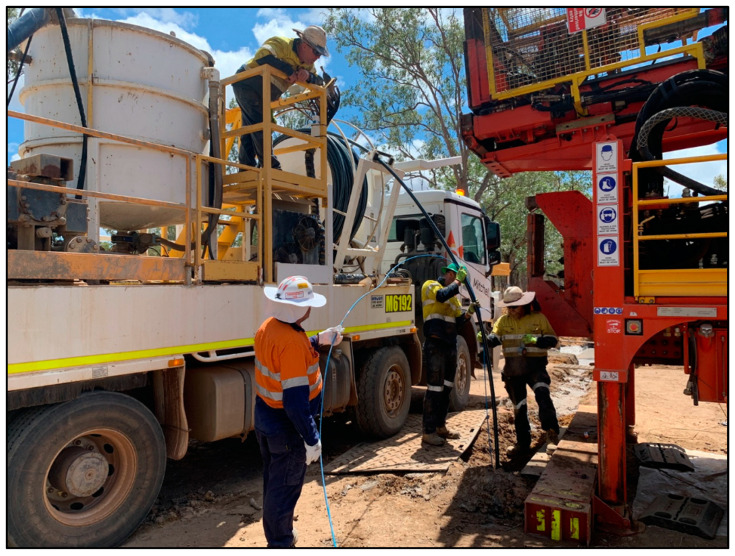
Installation of the fibre optic cable on a poly pipe to Borehole 2. The polyethylene pipe is attached to a grouting truck that was used to cement the borehole through the pipe.

The depth of both Boreholes 2 and 3 is about 400 m. The fibre-optic cables were installed in these two boreholes using slightly different methods. The cable in Borehole 3 was attached to a polyvinyl chloride (PVC) water-bore casing and lowered down the holes. The casing was then used to pump cement into the borehole to complete the installation. The fibre in Borehole 2 was installed using a 50 mm Blue Line HDPE pipe (high-density polyethylene pipe), to which a fibre-optic cable was taped (Figure 2). To ensure that the pipe reached the bottom of the hole, it was lowered with a non-metallic weight attached. The pipe was then used to pump cement into the borehole. The installation of the fibre into each of the boreholes took less than a day per borehole. 

**Figure 3:**
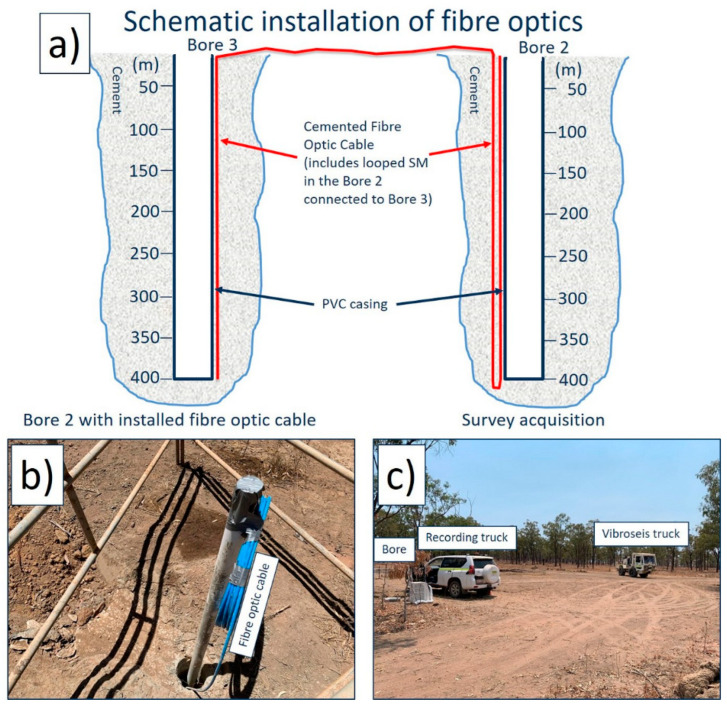
(**a**) Schematic picture of the fibre optics installation in the bores; (**b**) The wellhead of Borehole 3 with a coiled excess of the fibre optic cable; (**c**) The recording vehicle housing the fibre optic interrogator and a vibroseis truck during the survey acquisition at Borehole 2.

The choice of materials to deploy the cable and cement the holes was to ensure no metallic components were installed in the subsurface that could interfere with future mining operations. This included the turnaround made for the cable deployed in Borehole 2 that allowed covering the borehole twice by a fibre path and establishing the connection with Borehole 3 (Figure 3a), as well as the attenuator for the bottom of Borehole 3 to avoid the strong reflection of laser light from the end of the cable. The 5 mm armoured cable connecting the two boreholes was buried about 10 cm below the surface at track crossings to protect it from traffic. Figure 3 shows the schematics of fibre-optic connections and pictures from the survey (the wellhead of Borehole 2, the recording vehicle and a vibroseis truck).

The acquisition was conducted using 26,000 lbf peak-force vibroseis trucks (Figure 3) transmitting a 10 s linear 6–160 Hz sweep. Shot line spacing was 100 m, and the distance between shot locations on a line was 10 m. The installed optical cables contain single-mode fibres, which were used for DAS VSP acquisition. DAS data were recorded using a dual-pulse Fotech Theta interrogator with the following recording parameters: sampling rate of 1 ms, channel separation of 0.68 m, pulse repetition frequency of 30 kHz, pulse width of 150 ns and a gap of 60%. These settings are equivalent to a gauge length of about 12 m. The total length of the interrogated fibre-optic cable was about 2400 m. The interrogator was recording continuously, with a GPS time stamp for each record. During processing, the data were matched to actual sweep records using the recorded vibroseis time breaks. 

## 3. Synthetic Study

Processing and imaging seismic data in geological settings with such a high-velocity contrast is usually quite challenging. To determine the optimal processing parameters and to aid in the interpretation of the results, we performed acoustic synthetic modelling of the expected seismic response. To create synthetic seismograms, we needed a subsurface model incorporating P-wave velocity (VP) and density. These parameters are ideally obtained from borehole logs, and indeed, the boreholes that we used for the data collection were logged accordingly. Unfortunately, however, the holes were cased to a depth of approximately 200 m, and thus VP measurements were available only for the deeper parts of the boreholes, and we had to infer the values from the measured density logs. 

**Figure 4:**
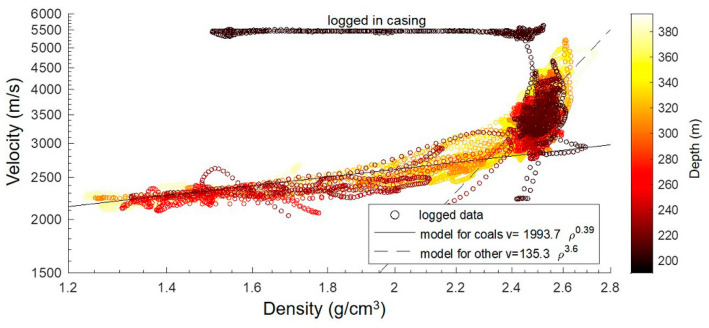
Log/log plot of the estimated relationship between P-wave velocity and density based on the log data from the bottom of the holes. The constant velocity datapoints correspond to the velocity of the steel casing, as indicated by the dark colour corresponding to the shallow depths. The datapoints were fitted by two curves (solid and dashed lines in the log/log plot) for the different density ranges, since it is clear that one line would not produce a good fit.

There are several empirical relationships between density and VP commonly used for sedimentary rocks [23,24]. However, such empirical estimates are too general, and the results are usually inconsistent with borehole logs in a specific area, which results in adapting the parameters in the formulas for the specific case. To find the best-fitting relationship between the density and VP, we used the available logs to cross-plot these quantities (Figure 4). There are some measurements logged within the cased part of the bores that are present on the plot. They fall around 5500 m/s of the velocity value and a wide range of density values (points are labelled in Figure 4). These datapoints were excluded from the following fitting with empirical property trends.

**Figure 5:**
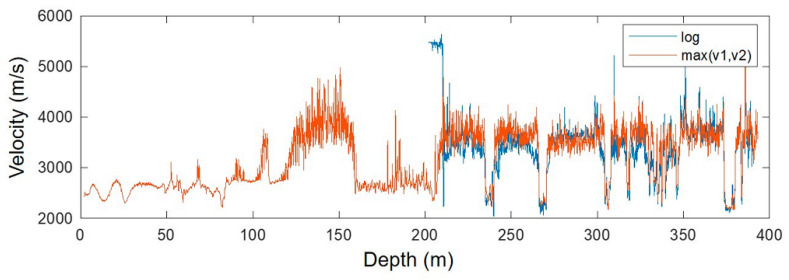
P-wave velocity computed from the density log compared to the logged sonic velocity in Borehole 1.

Since we could not fit the data using only one empirical relationship, we split the data into two sets, for which we found the best exponential fits for these two separate parts of the data. These two fits were separated by a density of 2.4 g/cm^3^, at which the behaviour of the relationship changes. The resulting VP-density relationship is a combination of the two models, which are shown in Figure 4 (solid and dashed lines in the log/log plot). Figure 5 shows the final velocity profile compared to the logged sonic velocities below 200 m in Borehole 1—the match is sufficient for the modelling of the seismic response. A combination of the reconstructed plus measured velocities was then used to build a horizontally layered model for the numerical simulation of the seismic response. 

The seismic response was modelled using OASES, a computer code for modelling full-wave seismo-acoustic propagation in horizontally layered environments [25]. The modelling was carried out for a 2D geometry with a 400 m vertical borehole with 1 m spaced sensors and sources from 5 m to 850 m away from the borehole with 1 m spacing. Both vertical and horizontal components of particle velocity produced by waves generated by a vertical point force were generated for each receiver location. The complex geology of the area results in a complex seismic wavefield. Because the focus was on processing the reflected P-waves of the field data, understanding the effects of P-wave multiples on imaging was of interest, and as such, only acoustic modelling was used. 

The synthetic dataset was composed of the computed traces corresponding to the offsets taken from the exact geometry of the field experiment using the location of Borehole 2. The simulated waveforms essentially form a regular grid relative to offset (5 to 850 m, step 1 m) and elevation difference (0 to 400 m, step 1 m) between source–receiver pairs. Thus, for each of the field source–receiver pairs, a corresponding synthetic seismic trace was assembled using bilinear interpolation of the nearest synthetic traces based on the offset and elevations. Therefore, the simulation results mimic the exact geometry of the field experiment.

Then the synthetic particle velocity data were converted into the strain rate, which is the native measurement of this DAS system [26]. Firstly, synthetic vertical and horizontal components of the wavefield were used to calculate the particle velocity along the borehole, accounting for the borehole geometry. Secondly, the data were differentiated along the length of the borehole. The obtained wavefield represents the strain rate, and thus the synthetic data become fully comparable with the field dataset. 

**Figure 6:**
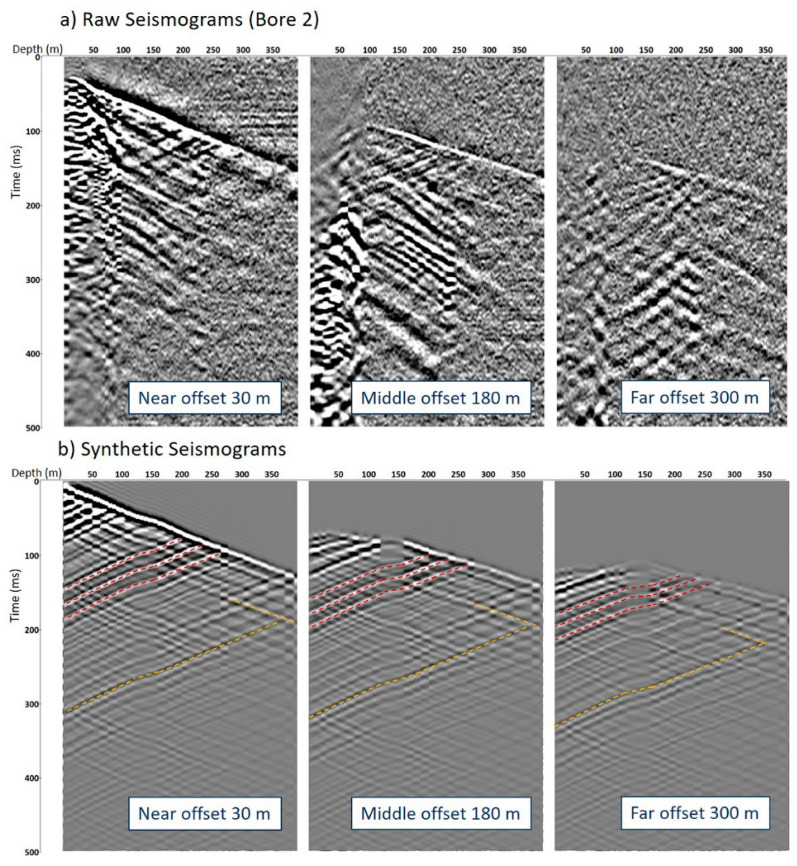
Examples of field raw and synthetic seismograms: (**a**) raw common source gathers at 30, 180 and 300 m offset from Borehole 2; (**b**) respective synthetic common source gathers at 30, 180 and 300 m. Red dashed lines indicate primary reflections; yellow dashed lines indicate multiple reflections.

Figure 6 shows examples of the field and synthetic DAS VSP data for three shot locations. The depths of the key seismic reflectors are positioned correctly in accordance with the logs. It can also be observed that the VP model overestimates velocities in the shallow part of the section, resulting in earlier arrivals of the direct wave. A better match between the synthetic and field data can be achieved if the model is updated accordingly. However, given the relatively low quality of the field seismic data and the lack of velocity logs available in the shallow part of the borehole, an adequate update of the high-resolution velocity model is challenging. 

**Figure 7:**
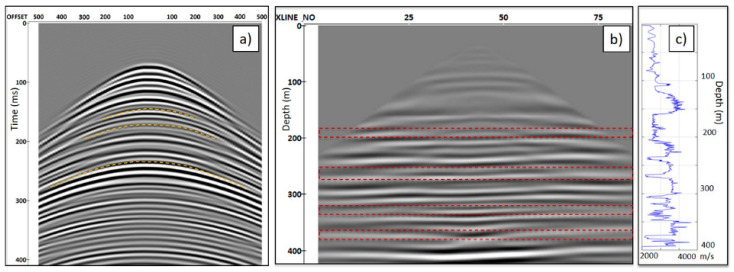
(**a**) The synthetic common receiver gathers at 200 m depth for the source line 5352. The yellow dashed lines indicate multiples; (**b**) a section of the migrated 3D synthetic volume. The artefacts that originated from multiples are highlighted by red dashed polygons; (**c**) P-wave velocity computed from the density log.

Even though the simulated seismic response does not fully match the field data, it gives a clear indication of the challenges that may be encountered in processing the seismic data. The synthetic modelling shows that the presence of numerous strong reflectors (red dashed lines in Figure 6b) results in the generation of numerous multiples (yellow dashed lines in Figure 6b), which affect the imaging unless attenuated. Figure 7a shows the common receiver gather for the channel at 200 m depth after separation of the upgoing wavefield, which displays several multiples (dashed yellow lines). The multiples strongly affect the migrated image by producing lens-type artefacts, as shown in Figure 7b. Therefore, the synthetic modelling reveals an important issue that should be accounted for to correctly process and interpret the data in coal seam geological settings.

## 4. Processing of Field 3D DAS VSP Data

**Figure 8:**
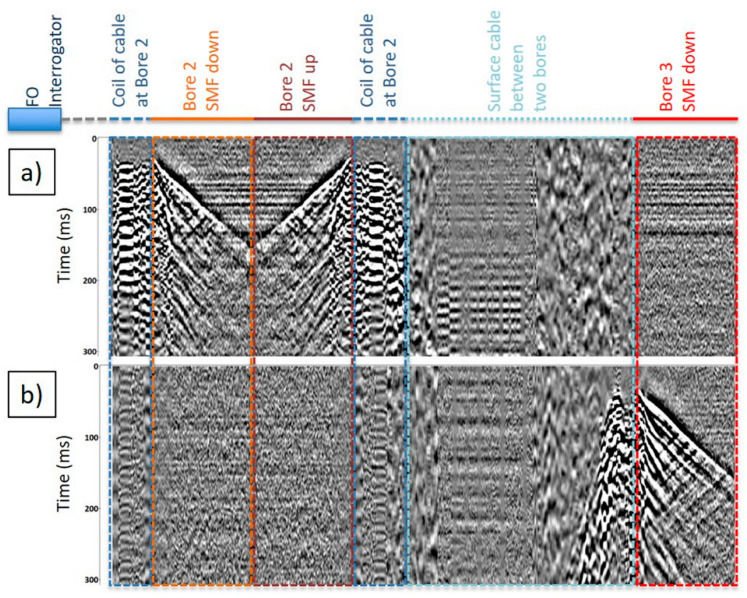
Examples of raw DAS data. Raw seismograms recorded with the continuous fibre-optic connecting Boreholes 2 and 3 to their respective nearest offset: (**a**) shot point nearest to Borehole 2 (~30 m); (**b**) shot point nearest to Borehole 3 (~30 m).

In total, 1647 source points were included in the analysis. Figure 6b and Figure 8 show examples of the raw data records. Figure 8 displays raw seismograms from the continuous fibre connecting both Boreholes 2 and 3 with marked segments of the cable. The two displayed seismograms are for the shot locations nearest to the two boreholes. Figure 6b shows panels of raw DAS seismic records at various offsets for Borehole 2 data. Strong reflections are observed in the raw records and are mainly related to the high velocity contrast of basalts and coal seams within the geological section. One can also observe the deterioration of the data quality with offsets, which is related to the directional sensitivity of fibre optics and partially to the quality of the cable coupling with the cement.

To process the data, first the geometry was assigned to the acquired datasets by matching the GPS time stamps of the recorded files with time breaks logged by a surface seismic system received from the vibroseis trucks’ decoders at each shot location. Next, all the data were loaded into the RadexPro v2021.3 processing software. Data were organised in two individual datasets for Borehole 2 and Borehole 3 and migrated separately. The same processing workflow was used for both datasets (Table 1). The channel–depth relationship of the optic channels was calibrated by pinpointing specific positions on the cable at known locations in the boreholes (such as the wellhead and bottom of the hole). Noisy traces and bad records were removed. Data were resampled to a 1 m depth interval (traces within 1 m windows were stacked). Specifically, for Borehole 2 data, to increase SNR, both directions (down and up) of the fibre-optic cable were also stacked (channels corresponding to the same depth were mathematically summed). Velocity was estimated using one of the closest shot points, which is a zero-offset geometry, for Borehole 2 and Borehole 3 independently. 

**Figure 9:**
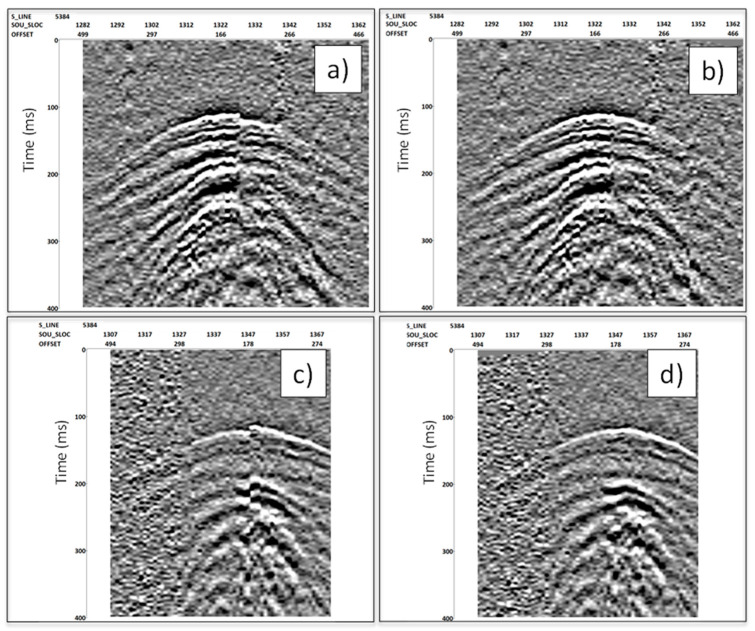
Example of the application of a model-based static correction for both boreholes at 200 m depth. Source line 5384. (**a**) Data from Borehole 2 before and (**b**) after static was applied; (**c**) Data from Borehole 3 before and (**d**) after static was applied.

As data quality deteriorated relatively rapidly away from the boreholes, it was not possible to pick first breaks along the entire record for offsets beyond ~150 m. As such, to compensate for source statics and to broaden the spectrum of the wavelet, deterministic deconvolution had to be replaced with the following alternative approach: Static corrections were estimated using a model-based approach; travel–time curves for the direct P-wave were calculated using ray tracing and the obtained velocities for a chosen depth (with a high SNR of the first breaks across a large offset range). The first breaks were then picked for the same depth and offset range, and the difference was attributed to the source static shifts and applied to the data. Figure 9 shows examples of the application of static corrections for data from both boreholes, and it demonstrates a clear improvement in the continuity of the interfaces.

**Figure 10:**
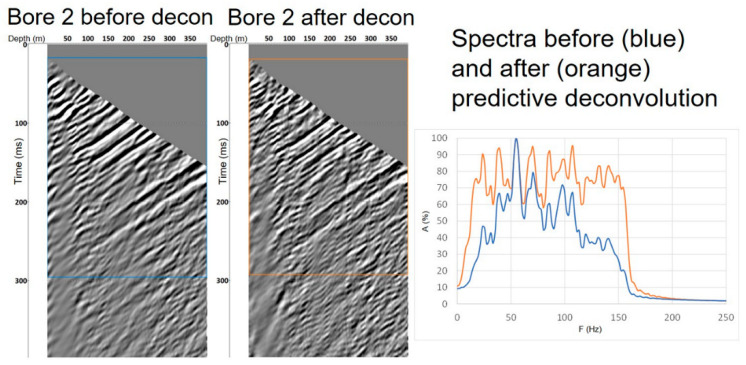
Example of the wavefield separation for Borehole 2 data (left panels show data after wavefield separation) and application of non-stationary predictive deconvolution. Right panel spectra before (blue) and after (orange) deconvolution.

The next step included the application of an FK filter and a 2D filter for the separation of an upgoing wavefield (reflections) for further analysis. To remove downgoing P- and S-energy, dedicated polygons were designed for the FK filter and applied in common shot gathers. The polygon focused on positive wave-numbers to suppress downgoing energy. Processing was followed by a 2D alpha-trimmed filter to suppress remaining noise (filter size of 11 traces and one sample at a time with 30% alpha-trim rejection). To exclude shear waves from the migration, we muted data below direct S-arrivals (muting parameters were estimated from direct S-wave travel times computed using ray tracing). The frequency spectrum was then evened and broadened by using non-stationary predictive deconvolution. Figure 10 displays an example of the application of deconvolution for the upgoing wavefield from the Borehole 2 dataset, with an evident increase in vertical resolution due to the broader spectrum.

**Figure 11:**
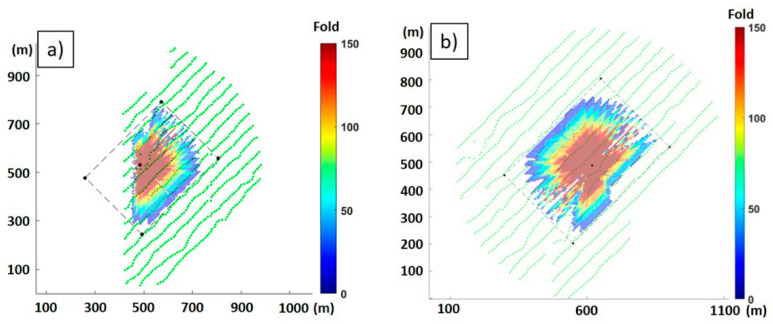
Fold maps at the target interval at the deepest coal seam of 380 m for the source point range selected for migration: (**a**) for Borehole 3; (**b**) for Borehole 2. Coordinate grids are relative and independent for each map.

The imaging was performed using an in-house 3D VSP Kirchhoff time migration code and a 1D isotropic velocity function obtained separately for each borehole. The algorithm was adapted to take into account the directional dependency of DAS amplitudes [20] by dividing the weights of the samples taken into migration by the cosine of the angle of the ray’s incidence to a borehole trajectory. The adapted migration also assumes straight ray paths. To simplify the quality control of the processing, the boreholes were imaged independently from each other on the common grid with a 6 m × 6 m × 1 m bin size. An amplitude correction for spherical divergence was applied before migration. To avoid introducing noise from low-quality gathers due to the directional variations in the sensitivity of DAS, an offset range for migration was limited to 500 m around the boreholes. The fold maps for the target horizon (~380 m) from the selected ranges of source locations for Borehole 2 and Borehole 3 are shown in Figure 11. 

**Figure 12:**
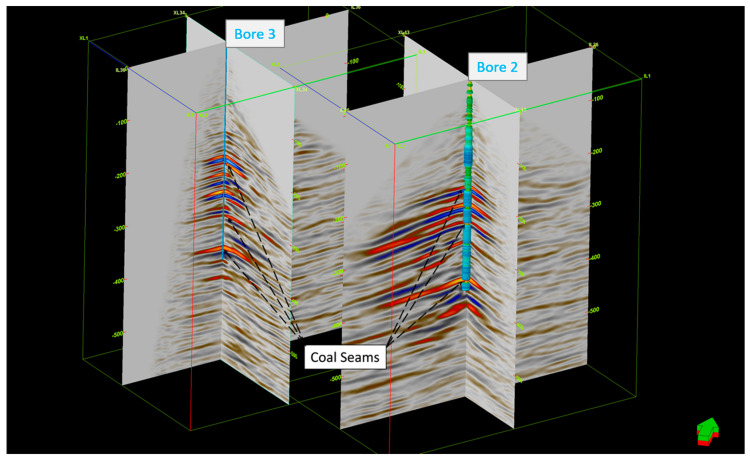
The 3D DAS VSP migrated volumes for Borehole 2 and Borehole 3. The density log is overlayed on the Borehole 2 seismic volume.

**Figure 13:**
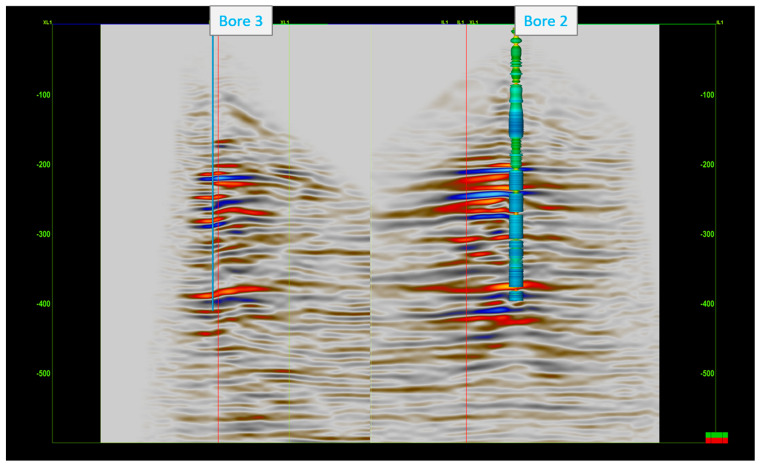
The 2D transect across the two boreholes. Density log is overlayed on the Borehole 2 seismic volume.

Figure 12 shows the migrated DAS 3D VSP volumes for both bores. The applied DAS methodology produced clear images of the target coal seam formation (around 380 m depth). As can be seen in Figure 12, which also shows the density log in Borehole 2, the 3D cube is consistent with the logs. The coal seams on the log are related to the low density readings, which are shown in yellow. The target deep coal horizon is illuminated and easily traced around the boreholes in both volumes. The upper coal seams are apparent as well and consistent across the volumes. Figure 13 shows a 2D transect across both boreholes. The main coal seams are consistently imaged within both volumes. Unfortunately, they intersect at the area of a low fold, which makes collaborative interpretation somewhat difficult. 

## 5. Discussion

Despite the encouraging results of the trial, there remain several challenges that require further study. It would be ideal to migrate data for both bores simultaneously, but there are several reasons why it is currently preferable to image and analyse the datasets individually. 

First, the data at mid- and far-offsets are quite noisy. This prevented us from estimating the arrival times of direct P-waves across the survey, which could be used to account for azimuthal anisotropy [10] and help to build a more accurate and consistent velocity model for combined imaging. In this study, velocity models were derived separately for each borehole. The introduction of an engineered fibre with enhanced backscattering properties for such deployments would substantially increase the SNR [12] of recorded data. This will likely soon be a common practice as the DAS technology is rapidly evolving. 

An additional weighting of amplitudes in the applied Kirchhoff migration was introduced, which compensates for the directional sensitivity of the fibre sensors. This brings some additional noise to the migrated images, especially from large offsets, and this issue needs to be more carefully addressed in the future. Scrutiny of amplitude compensation accounting for DAS VSP geometry is required to avoid artefacts while doing concurrent migration of multi-borehole data.

Other noise components that require additional attention and separate studies are source-generated and converted S-waves, as well as surface-related and interbed multiples. At this stage, a simple mute of most of the S-wave energy below the direct S-wave arrivals was used, but a more prudent approach based on noise modelling should be investigated to reduce such contamination. Geological settings in the area include quite a few formations with high contrast in velocities compared to surrounding rocks, such as coal seams themselves and the basalt cover. This inevitably leads to the generation of multiples, as demonstrated in this synthetic study. Finding the most appropriate solution to suppress these multiples is another future research direction.

Despite these challenges, in this pilot study, the benefits of downhole DAS seismic measurements are apparent. Furthermore, permanently deployed downhole DAS cables create additional value by turning a borehole into a seismic sensing array that can be utilised for mine safety monitoring. Designing in advance which fibre types (multimode, single-mode, tight-buffered, etc.) to include in a cable for installation allows bringing extra advantages like implementing simultaneous measurements of multiple properties such as strain and seismic events, which can be further used for subsurface characterisation of the physical properties and mine monitoring. The presented study demonstrates great potential for incorporating seismic fibre-optic technology into the exploration and production workflows of the coal industry. The quality of the final seismic 3D images demonstrates a high value of information obtained using the DAS technique.

## 6. Conclusions

A DAS 3D VSP field study for coal seam exploration produced a high-quality 3D seismic image that illuminates the target coal seam in the vicinity of Borehole 2 and Borehole 3. The experimental installations of fibre optics in several bores demonstrated that this technology could be easily included in the standard exploration drilling workflow with substantial cost efficiency. The advantages of DAS technology, in conjunction with downhole seismic methodology, permit us overcome difficulties in imaging the subsurface formations below the basalt cover by having multiple seismic sensors underneath it. Instrumenting the exploration bores with DAS sensors allowed for a clear 3D image and a reduction in the duration of the whole field campaign quite considerably. 

Several aspects of data analysis that need some improvement were identified, and some of them were addressed. Data acquired with fibre-optic sensors should be treated carefully. Here, a modified standard Kirchhoff time algorithm was used to account for the DAS’s distinct directivity. Additional studies are required in data analysis to better suppress S-waves and multiples from the upgoing wavefield gathers. This synthetic study significantly improved our understanding of the wavefield components using real data and helped validate approaches to processing.

The permanently deployed fibre optics create a seismic monitoring array in each of the bores. That considerably increases the value of the deployed DAS cables, as they can be used for further monitoring of the mine operations as well as for measurements of other properties like temperature, strain and passive seismic events. Considering the economic and operational advantages of DAS over conventional receivers and the achieved quality of 3D DAS VSP imaging, fibre-optic technology becomes appealing as a versatile and cost-effective tool for the coal industry.

## Figures and Tables

**Table 1:** 3D DAS VSP processing flow.

Data Preparation	Converting DAS records to standard SEGY files.
Data Input and Geometry	Assigning each FFID with its respective source coordinates, source line and shot number; assigning depth to DAS channels.
Data Editing	Removing noisy traces.
Static Correction	Applying model-based static corrections.
Amplitude Correction	Correction for spherical divergence.
Predictive Deconvolution	Applying non-stationary predictive deconvolution.
Velocity Analysis	Estimating the velocity function from ZVSP.
Wavefield Separation	In common shot-gathers, an FK filter is used in dedicated polygons to remove downgoing P- and S-waves, followed by a 2D alpha-trimmed filter. Mute energy above direct P arrivals and below direct S arrivals.
Migration	Isotropic velocity model, Kirchhoff migration central dip = 0, dip range = 7 degrees. Offset limited by 500 m.

## Data Availability

Restrictions apply to the availability of the DAS data. These data were obtained at the Anglo American exploration site within the MinEx CRC project. Data are available from the authors with the permission of the MinEx CRC project and at Anglo American.
